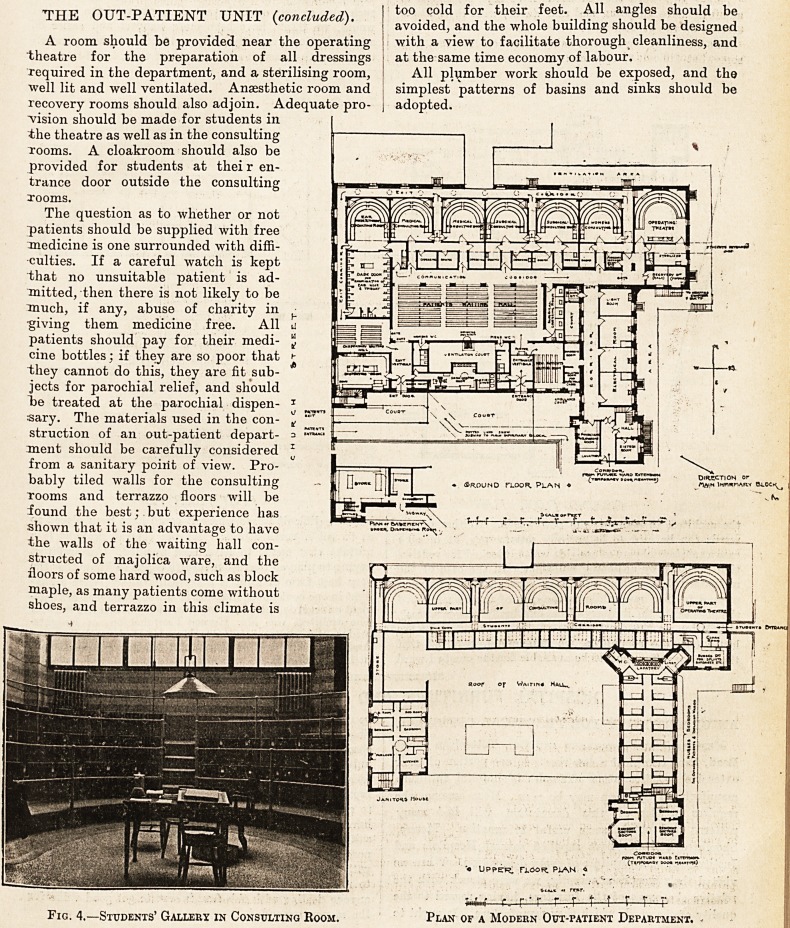# The Units of General Hospital Construction

**Published:** 1907-06-29

**Authors:** 


					Jose 29, 1907. THE HOSPITAL. 351
HOSPITAL ADMINISTRATION.
(/K CONSTRUCTION AND ECONOMICS.
THE UNITS OF GENERAL HOSPITAL CONSTRUCTION.
THE OUT-PATIENT UNIT
(concluded).
A room should be provided near the operating
"theatre for the preparation of all dressings
required in the department, and a sterilising room,
well lit and well ventilated. Anaesthetic room and
recovery rooms should also adjoin. Adequate pro-
vision should be made for students in
the theatre as well as in the consulting
rooms. A cloakroom should also be
provided for students at thei r en-
trance door outside the consulting
xooms.
The question as to whether or not
patients should be supplied with free
medicine is one surrounded with diffi-
culties. If a careful watch is kept
that no unsuitable patient is ad-
mitted, then there is not likely to be
much, if any, abuse of charity in
giving them medicine free. All
patients should pay for their medi-
cine bottles; if they are so poor that
they cannot do this, they are fit sub-
jects for parochial relief, and should
Toe treated at the parochial dispen-
sary. The materials used in the con-
struction of an out-patient depart-
ment should be carefully considered
from a sanitary point of view. Pro-
bably tiled walls for the consulting
rooms and terrazzo floors will be
found the best; but experience has
shown that it is an advantage to have
the walls of the waiting hall con-
structed of majolica ware, and the
floors of some hard wood, such as block
maple, as many patients come without
shoes, and terrazzo in this climate is
too cold for their feet. All angles should be
avoided, and the whole building should be designed
with a view to facilitate thorough cleanliness, and
at the same time economy of labour.
All plumber work should be exposed, and the
simplest patterns of basins and sinks should be
adopted.
TpTT-n /^ttt t> a ttpxthh / 7 ji j\ too cold for their fecfc# A.11 cHi^Igs should bo
THE OUT-PATIENT UNIT (concluded). avoided, and the whole building should be designed
A room should be provided near the operating with a view to facilitate thorough cleanliness, and
"theatre for the preparation of all dressings at the same time economy of labour.
required in the department, and a sterilising room, All plumber work should be exposed, and the
well lit and well ventilated. Anaesthetic room and simplest patterns of basins and sinks should be
recovery rooms should also adjoin. Adequate pro- adopted.
vision should be made for students in
the theatre as well as in the consulting
rooms. A cloakroom should also be
provided for students at thei r en-
trance door outside the consulting
xooms.
The question as to whether or not
patients should be supplied with free
medicine is one surrounded with diffi-
culties. If a careful watch is kept
that no unsuitable patient is ad-
mitted, then there is not likely to be
much, if any, abuse of charity in ^
giving them medicine free. All ti
patients should pay for their medi- ?
cine bottles; if they are so poor that *?
they cannot do this, they are fit sub-
jects for parochial relief, and should
Ibe treated at the parochial dispen- 1
sary. The materials used in the con- ^
struction of an out-patient depart- =
inent should be carefully considered 1
from a sanitary point of view. Pro-
bably tiled walls for the consulting - III . srouno floor, plan ? ?> oioe^,'
rooms and terrazzo floors will be Ei ? ?
iound the best; but experience has Fir?* '"""-"Cx T f . r > > ,. ,r 1
shown that it is an advantage to have
the walls of the waiting hall con- i ~~ 1
structed of majolica ware, and the u ?1   ? n \ mm >mm
floors of some hard wood, such as block **'<>??' * T
maple, as many patients come without ' j (uv!' I ull pu?i it
shoes, and terrazzo in this climate is ij III ~ jj - ? Li] j.
Upper, Fj-Oor. Pjan j
tiAit ?r rr*r.
w r. r t rr f T T T r
Fig. 4.?Students' Gallery in Consulting Room. Plan of a Modern Out-patient Department.

				

## Figures and Tables

**Fig. 4. f1:**